# Psychosocial Stress in the Chinese Community: Speech Analytics Through Linguistic and Acoustic Fusion Using Machine Learning

**DOI:** 10.2196/91138

**Published:** 2026-05-29

**Authors:** Amanda M Y Chu, Benson S Y Lam, Jenny T Y Tsang, Agnes Tiwari, Jacky N L Chan, Mike K P So

**Affiliations:** 1 Department of Social Sciences and Policy Studies, The Education University of Hong Kong, Tai Po, Hong Kong, China; 2 Department of Mathematics, Statistics and Insurance, The Hang Seng University of Hong Kong, Shatin, Hong Kong, China; 3 School of Nursing, Tung Wah College, Homantin, Hong Kong, China; 4 School of Nursing, Hong Kong Sanatorium and Hospital, Hong Kong, China; 5 Department of Information Systems, Business Statistics and Operations Management, The Hong Kong University of Science and Technology, Clear Water Bay, Hong Kong, China, 852 2358 7726

**Keywords:** caregivers, digital health, psychosocial health, speech analytics, text analytics

## Abstract

**Background:**

Family caregivers experience significant stress due to intensive caregiving activities, making them highly susceptible to adverse psychosocial health conditions. Early detection of this stress is crucial for timely interventions to prevent disease progression and long-term disability.

**Objective:**

This study aimed to develop and validate the Linguistic and Acoustic Speech Analytics Program, a novel machine learning approach capable of providing a fusion analysis of linguistic and acoustic speech features to enhance the effectiveness of psychosocial stress assessment.

**Methods:**

This quantitative study analyzed speech data collected from 100 Chinese family caregivers. Participants responded to 12 open-ended questions, and their voices were recorded for linguistic and acoustic feature extraction. Various machine learning classifiers, including support vector machine, were developed to process speech data. A key methodological step was the application of an orthogonalization procedure to decorrelate acoustic features from linguistic features before fusion analysis. The classifiers were then trained to evaluate psychosocial stress levels based on the processed and fused linguistic and acoustic speech features. Model performance was measured using receiver operating characteristic-area under the curve, *F*_1_-score, and accuracy.

**Results:**

The linear support vector machine model emerged as the top performer, achieving a receiver operating characteristic-area under the curve of 78.28%, an *F*_1_-score of 75.27%, and an accuracy of 73%. These results demonstrate the model’s strong capability in identifying stressed participants based on their speech. Critically, the fusion of linguistic and acoustic features significantly outperformed models using either feature type alone. Furthermore, the orthogonalization procedure proved essential, as decorrelating features before fusion markedly enhanced classification accuracy compared to using non-orthogonalized features.

**Conclusions:**

This study demonstrates that fusion analysis of linguistic and acoustic features effectively identifies psychosocial stress among family caregivers. It also emphasizes the importance of proper feature processing when combining multiple features extracted from the same audio sample. These findings provide valuable insights for developing machine learning models for psychosocial stress assessment and addressing various psychosocial conditions in different contexts, supporting population mental health management.

## Introduction

Psychosocial wellness, encompassing the intricate interplay of mental, emotional, social, and spiritual well-being, is essential for overall health. However, the increasing pressures of modern life expose individuals to various stressors, significantly contributing to a rise in psychosocial conditions, which involve a broad category of mental health disturbances and social behavioral patterns that can impair daily functioning [1]. The World Health Organization (WHO) reports that one in eight people globally live with a mental health condition, with anxiety and depression being the most prevalent [2-4]. If left unaddressed, these issues can escalate into severe mental disorders, leading to significant disability. Mental disorders account for 16% of global disability-adjusted life years and impose an annual economic burden of approximately US $5 trillion (2019 dollars) [5], highlighting the profound effects of psychosocial conditions on individuals, families, and society.

Early detection and intervention are crucial to the effective treatment of psychosocial conditions [6]. However, 75% of individuals in need, particularly in low- and middle-income countries, do not receive adequate interventions [3]. A major obstacle is the lack of easily accessible and efficient assessment services. Currently, the gold standard assessment method involves comprehensive individual interviews conducted by specialists using validated questionnaires, which is time-consuming and resource-intensive [7]. Furthermore, these assessments rely on clients’ responses that can be subjective and unreliable. For instance, public stigma surrounding psychosocial conditions may lead clients to provide socially acceptable answers rather than truthful ones [8,9]. Individuals may also lack complete self-awareness, making accurate self-reporting challenging [10]. Consequently, identifying subtle signs of psychosocial conditions often relies heavily on the assessor’s experience, impacting both the efficiency and accuracy of evaluations. Thus, there is an urgent need to develop an automated, scalable system that enables objective and efficient assessment of psychosocial conditions.

Speech, as an information-rich signal conveying unique thoughts and emotions, can serve as a behavioral marker with which to assess psychosocial health [11]. Speech has long been recognized as a process requiring complex motor coordination, involving more than 100 muscles, and supported by an extensive network of brain regions that handle auditory, somatosensory, and visual information, along with language comprehension and production [12]. Hence, spoken communication is a valuable window into the mind, creating opportunities for technologies that capture and process speech in order to evaluate psychosocial health. Recent advances in digital technologies and machine learning provide a foundation for developing automated systems to assess psychosocial health through speech analytics [6].

Speech consists of linguistic and acoustic features, both known to indicate mental disorders [13]. Linguistic features include the frequency of specific words and phrases, often analyzed through text extraction, topic modeling, and word embeddings. The elevated use of certain terms has been identified as a marker of anxiety, depression, and suicidal ideation [14-16]. Acoustic features encompass various voice quality characteristics, such as pitch, intensity, speech rate, prosody, and jitter. Research indicates that patients with depression tend to exhibit a lower pitch, more monotonous speech, reduced sound intensity, and slower speech rates [17,18]. While linguistic and acoustic features can reflect an individual’s psychosocial status, many reported speech analytics systems focus on only one feature. Furthermore, despite the insights gained from speech analytics in assessing mental health conditions, few studies have specifically explored its application in the assessment of psychosocial stress.

Stress is a common initial indicator of psychosocial issues and a known risk factor for numerous mental and physical health problems [19,20]. Identifying stress is therefore crucial for early intervention and prevention of severe mental disorders. While speech analytics has shown potential for detecting stress, most studies focused on analyzing either linguistic or acoustic features alone, resulting in suboptimal accuracy for practical applications in psychosocial services [14,21].

Recent research suggests that the fusion analysis of linguistic and acoustic features can enhance mental health assessments, including the identification of suicidal ideation and depression [11,22]. However, existing studies often rely on voice recordings obtained by directly inquiring about sensitive topics. This approach can lead to misleading results, as participants may be reluctant to disclose their true psychosocial status due to social stigma. To overcome this, we conducted this study in a real-world community setting, where social workers asked participants nonsensitive and open-ended questions about family life, allowing them to express their true feelings and reducing the risk of response bias. To the best of our knowledge, this is the first study of its kind conducted within a Chinese community.

To address the challenge of the automatic stress-level classification problem, we developed the Linguistic and Acoustic Speech Analytics Program (LASAP) to provide a comprehensive fusion analysis of both linguistic and acoustic features to improve psychosocial stress classification. This study applies the LASAP to detect psychosocial stress among family caregivers—a group particularly vulnerable to significant stress due to physical, emotional, and financial challenges associated with family caregiving. Recognizing signs of stress and implementing supportive measures can help caregivers maintain their psychosocial health and overall quality of life.

Another challenge in current fusion studies is the lack of robust methods for processing linguistic and acoustic features before they are combined. This step is crucial because features extracted from the same speech sample are naturally correlated, and using them together without proper processing can lead to information redundancy and an increased risk of model overfitting [23]. For instance, when an individual states, “I feel overwhelmed and stressed every day,” the words “overwhelmed” and “stressed” serve as linguistic indicators of stress. Simultaneously, the acoustic features extracted might reflect a higher pitch when saying “overwhelmed” and a louder volume when emphasizing “stressed,” both conveying emotional intensity. This natural correlation between linguistic and acoustic features may hinder subsequent analyses. If these features are analyzed together without decorrelation, the emotional state indicated by both might be overemphasized, potentially leading to overfitting in the developed automated system [24]. Therefore, we implemented a novel orthogonalization procedure to decorrelate the acoustic features from the linguistic features [25].

Using well-processed features, the LASAP successfully distinguished between family caregivers with different stress statuses. By integrating both linguistic and acoustic features, it achieved higher accuracy in stress detection compared to analyses of either feature alone. Additionally, the LASAP streamlines data collection by asking a limited number of targeted general questions, minimizing interview time, and improving assessment efficiency. This automated program not only enables accurate and efficient evaluations but also holds promise for delivering accessible and affordable psychosocial services through digital health technologies. This research highlights the potential of fusion analysis to enhance the accuracy of early psychosocial assessments and underscores the importance of proper feature processing for effective analysis.

The goal of the LASAP differs significantly from that of our previously developed method, the Automatic Speech Analytic Program (ASAP) [14]. The LASAP aims to predict an individual’s stress level through the fusion analysis, which combines both linguistic and acoustic features, and the orthogonalization procedure. This approach involves building a machine learning classifier trained on a set of labeled data and evaluated on a separate, unseen testing dataset. In contrast, the ASAP utilizes a clustering technique to split a group of individuals into low-stress status and high-stress status. However, due to the splitting nature of the clustering technique, the ASAP requires all individuals to be present at the start of the analysis. If a new, unseen individual is introduced after the analysis has begun, that individual cannot be categorized. Additionally, the ASAP relies solely on linguistic features for its analysis and does not incorporate acoustic features.

## Methods

### Study Design and Participants

Family caregivers from diverse backgrounds were recruited from a nonprofit organization providing integrated family and community services in Hong Kong. A total of 100 family caregivers were approached and recruited by registered social workers, who explained the study and obtained signed informed consent from participants. Participation was entirely voluntary.

Stress levels of the 100 caregivers were assessed using the validated Caregiver Burden Inventory (CBI) [26]. The CBI is a 24-item self-report scale that evaluates caregiver stress through a multidimensional approach, consisting of 5 subscales: time dependence, developmental, physical, social, and emotional burden. This study adopted a validated Chinese version of the CBI, which demonstrated a Cronbach α of 0.95 [27]. Participants assessed each item using a 5-point Likert scale, where “0” indicates “not at all descriptive” and “4” indicates “very descriptive.” Higher scores reflect greater caregiver stress. A total score of 36 or lower indicates low levels of stress, whereas a score above 36 indicates high levels of stress. Ultimately, 44 caregivers were classified as low stress and 56 as high stress. Qualified social workers from the nonprofit organization, who were familiar with the participants, agreed with the classification based on their understanding of the participants.

In addition to completing the CBI, all participants were asked 12 open-ended questions about their families and feelings. Their responses were recorded for training and testing the LASAP machine learning model. Existing literature suggests that open-ended questions provide more information than read speech tasks, and hence, they were adopted in this study [28]. The 12 questions, designed to explore family resilience, are listed in Table S1 in Multimedia Appendix 1. They cover 3 broad processes of Walsh family resilience theory: family belief systems, organizational patterns, and communication patterns. Family belief systems indicate the ability to overcome crises by finding meaning in adversity, maintaining a positive outlook, and fostering spiritual beliefs. Organizational patterns reflect supportive family relationships that are flexible, connected, and accessible to social networks and economic resources. Communication patterns denote the capacity of family members to communicate effectively, ensuring clarity, open emotional expression, and problem-solving in challenging situations. Previous studies have shown that family resilience is closely related to caregiver stress, with higher levels of family resilience correlating with lower stress levels [29]. These nonsensitive questions allow participants to discuss their daily lives casually and express their true feelings, making them a less intrusive approach than directly querying about caregiver stress. Since family resilience is strongly correlated with stress levels, these questions can effectively reflect participants’ stress burdens [30]. From the responses of the 100 participants, we identified 53 keywords (representing 53 linguistic features) across 14 different topics (see Table S2 in Multimedia Appendix 1).

### Ethical Considerations

This project was approved by the Human Participants Research Panel of The Hong Kong University of Science and Technology (reference number 252). The research followed the Declaration of Helsinki ethical principles. All participants received detailed information about the study’s aims and procedures and provided informed consent. Participants had the right to withdraw from the study at any time, and participation was entirely voluntary. Data were anonymized, securely stored, and used solely for research purposes.

### LASAP Development

The LASAP was developed to optimize speech analytics with regard to assessing psychosocial stress. The program independently extracts linguistic and acoustic features from family caregivers’ speech. Linguistic features were derived by converting speech to text and analyzing word frequencies, with principal component analysis (PCA) applied to remove redundant information and retain meaningful features. A screening process identified 53 keywords linked to stress and family resilience topics based on the Walsh theory. Acoustic features were extracted using signal processing techniques via the openSMILE (audEERING GmbH) toolkit, generating 6503 features based on low-level descriptors (LLDs), such as “shimmer,” which measures vocal stability and emotional deficits. To prevent overlapping information between linguistic and acoustic features, an orthogonalization procedure was used to decorrelate them, ensuring independent contributions to the analysis. This approach improved the distinction between high-stress and low-stress samples, facilitating a more accurate assessment of caregiver stress and resilience.

### Linguistic Feature Extraction and Processing

**Figure 1.**
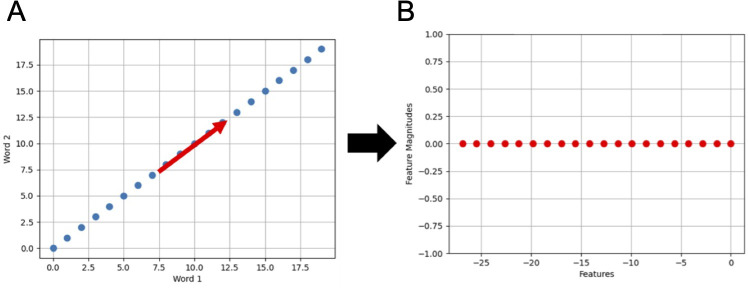
Illustration of the removal of redundant information using principal component analysis (PCA): (A) frequency counts of 2 words (before applying PCA) and (B) the results after applying PCA.

After collecting interview audio recordings from family caregivers, we converted their speech into text transcripts using the Google Cloud Speech application programming interface. The text was analyzed by counting the frequency of specific words and phrases. To eliminate redundant information, we applied PCA to remove words that carry the same sentiment, are frequently repeated, or are highly correlated with other words [31]. This process ensures that the dataset retains only unique and relevant linguistic features, enhancing the robustness and meaningfulness of subsequent analyses. Figure 1 demonstrates how PCA eliminates redundant information. In Figure 1A, the frequency counts of 2 words are shown. These words appear in all documents with the same frequency, resulting in a strong correlation between them. This correlation is visually represented as a diagonal line in the figure. PCA addresses this redundancy by identifying the diagonal direction as the primary axis (x-axis) and rotating the entire system to align with this new axis. This transformation is illustrated in Figure 1B, where the x-axis corresponds to the diagonal direction from Figure 1A. By doing so, PCA reduces the dimensionality of the representation, using only 1 dimension instead of 2 to capture the same information. This effectively removes redundant data.

We applied a screening process to select the 53 keywords from 2683 words that were extracted from all of the 100 text scripts. First, words or phrases were selected if they appeared 2 times or more and if they were related to a list of 14 family resilience topic categories identified through topic modeling [32]. After this screening process, 53 keywords or phrases were selected, related to the 14 different topics aligned with the 3 processes of the Walsh family resilience theory. This topic identification process facilitates a better classification of relevant words and contributes to an accurate assessment of caregiver stress, which is closely related to family resilience [30]. The frequency count of these 53 keywords among caregivers with high-stress or low-stress levels is listed in Table S2 in Multimedia Appendix 1 and serves as the linguistic features used in subsequent analyses.

### Acoustic Feature Extraction and Processing

We captured the acoustic features from the audio recordings using popular signal processing techniques, including the Fourier transform and spectral methods, implemented through the use of the Python package openSMILE [33]. Table S3 in Multimedia Appendix 1 presents a list of acoustic subsets adopted by INTERSPEECH 2016 ComParE (Computational Paralinguistics Challenge), built upon 65 LLDs that characterize the temporal and spectral properties of the acoustic signal [34]. Since the LLDs of an acoustic signal are themselves signals, we derive acoustic features by considering various mathematical and statistical characteristics of these LLDs. These characteristics include the simple moving average of an LLD, first-order derivatives, IQRs, minimum positive values, segment length SD, mean and SD of peak distances, mean peak relative height, flatness, rise time, skewness, left center time, and centroid. In total, we utilized 6503 acoustic features for this study.

**Figure 2.**
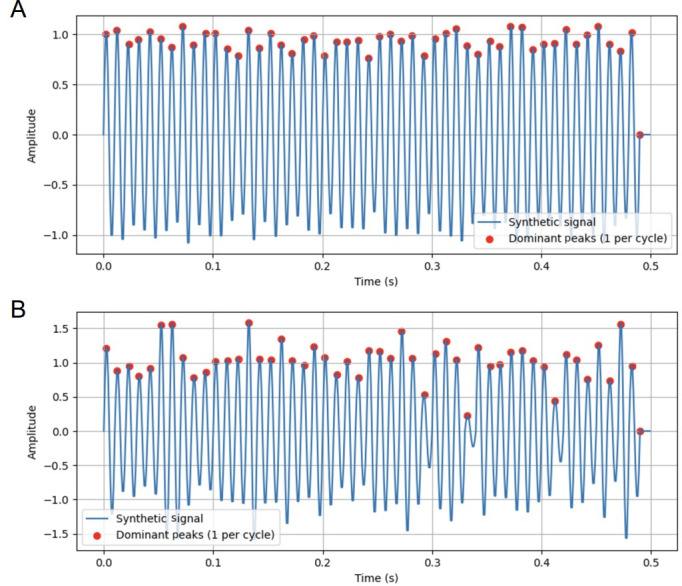
Illustration of shimmer, a low-level descriptor (LLD): (A) synthetic signal with low shimmer and (B) synthetic signal with high shimmer.

LLDs have been used to identify various emotional states. In this context, we focus on shimmer as an example. Shimmer quantifies the vocal stability of a sound wave over time [35]. Irregular vocal fold vibrations—often associated with emotional deficits—can lead to variations in shimmer. For instance, a depressed individual may speak with uneven loudness, reflecting instability in vocal amplitude. To illustrate this concept, we consider the synthetic signals as shown in Figure 2. The 2 figures represent wave cycles of speech, where the amplitudes of the wave shown in Figure 2A are more consistent compared to those in Figure 2B. Amplitude corresponds to the loudness of a voice; larger amplitudes indicate louder sounds. In the case of a depressed individual, uneven loudness may result in greater amplitude variations (as in Figure 2B). Shimmer provides a method to quantify this variation by measuring fluctuations in the peaks of the waveform. In doing so, shimmer effectively captures the stability of a voice, which can serve as an indicator of emotional deficits. However, in real-world scenarios, sound waves often vary in length, resulting in differing numbers of peaks and shimmer values. To standardize the number of features extracted, we calculate mathematical and statistical characteristics of the shimmer values, such as the minimum, maximum, quantiles, and other summary statistics.

Subsequently, we adopted the novel application of an orthogonalization procedure to decorrelate the acoustic features from the linguistic features. As we extracted both linguistic and acoustic features from the same audio recordings, they may contain overlapping information. For instance, when a family caregiver says, “I feel overwhelmed and stressed every day,” the emotion-related words “overwhelmed” and “stressed” are counted as linguistic features indicating stress. At the same time, the acoustic features extracted from the audio may include a higher pitch when saying “overwhelmed” and a louder volume when emphasizing “stressed,” which also convey emotional intensity. This phenomenon highlights a natural correlation between linguistic and acoustic features within a speech sample. If these features were analyzed together without decorrelation, the analysis might overemphasize the emotional state indicated by both. Therefore, we performed the orthogonalization procedure to decorrelate them. After orthogonalization, we found that the acoustic features of the high-stress sample are significantly diminished. This reduction may suggest that linguistic features carry substantial high-stress information, and orthogonalization removes this emotional information from the high-stress samples. Conversely, linguistic features seem to carry minimal low-stress information, resulting in a smaller impact of orthogonalization on the low-stress sample.

Orthogonalization is a mathematical process used to create a set of directions (or vectors) that are perpendicular to each other. This concept is illustrated in Figure S1 in Multimedia Appendix 1. The red dots represent an acoustic feature, while the green triangles represent a linguistic feature. As shown, these 2 directions are not perpendicular to each other; in other words, they are correlated and contain redundancy. To eliminate this redundancy, we apply a transformation to the acoustic feature, resulting in the blue line in Figure S1 in Multimedia Appendix 1. It can be observed that the blue line is now perpendicular to the green triangles. This transformation simplifies data analysis because each new feature represents a distinct direction of variation, free of redundancy.

**Figure 3.**
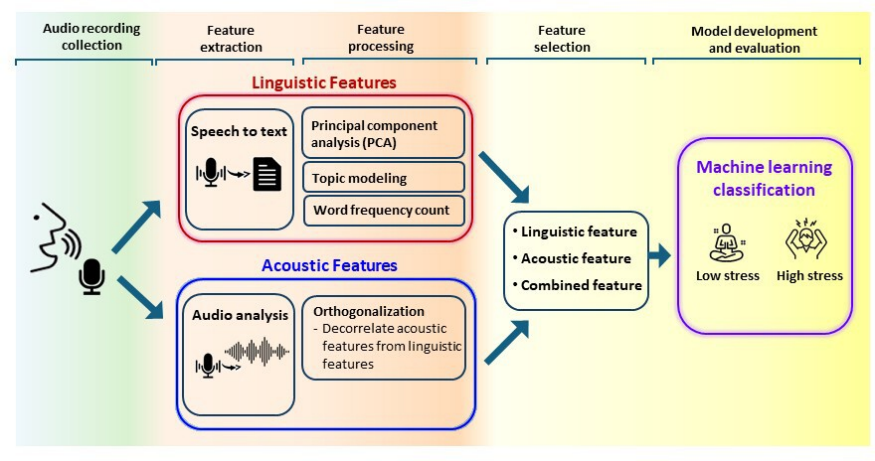
Overview of the Linguistic and Acoustic Speech Analytics Program (LASAP) pipeline.

An overview of the LASAP pipeline is illustrated in Figure 3.

### Machine Learning Classifier Models

Various machine learning classifier models, including linear support vector machine (SVM-linear), polynomial SVM (SVM-poly), radial basis function SVM (SVM-RBF), sigmoid kernel SVM (SVM-sigmoid), AdaBoost, ExtraTrees, k-nearest neighbor, and random forest, were trained using double cross-validation with a 10-fold outer cross-validation and a 5-fold inner cross-validation to differentiate psychosocial stress status based on the combined linguistic and acoustic features. Hyperparameters varied across settings, and those demonstrating the best performance in regard to the validation set are selected and listed in Table S4 in Multimedia Appendix 1.

We use *F*_1_-score, accuracy, and receiver operating characteristic-area under the curve (ROC-AUC) as evaluation metrics [36-38]. *F*_1_-score is the harmonic mean of precision and recall. The formula is as follows:

,(1) $$F_{1}\text{-}\text{score}\;=\frac{2TP}{2TP+FP+FN}\;\times \;100\%$$

where TP, FP, and FN are the true positives, false positives, and false negatives, respectively. In the *F*_1_-score, true negatives are omitted. Accuracy is the proportion of correct predictions among the total number of cases examined. The formula is as follows:

.(2) $$Accuracy=\frac{TP+TN}{TP+TN+FP+FN}\times 100\%$$

ROC-AUC reports the area under the receiver operating characteristic curve from prediction scores. The receiver operating characteristic curve plots the true positive rate (TPR) (=$\frac{TP}{TP+FN})$) against the false positive rate (=$\frac{FP}{TN+FP})$) at various threshold settings.

## Results

### Participants and Feature Characteristics

This study analyzed audio recordings from 100 family caregivers, with 44 classified as low stress and 56 as high stress. No statistically significant differences were observed between the 2 groups regarding demographic data.

The linguistic and acoustic features of the caregivers’ speech were extracted. The linguistic features used for machine learning analysis included frequency counts for 53 stress-indicating words, categorized into 14 different topics (Table S2 in Multimedia Appendix 1). The acoustic features consisted of 6503 characteristics, such as pitch, speech rate, and voice quality, organized into 65 subsets (Table S3 in Multimedia Appendix 1).

Following a common practice of training a machine learning classifier, a double cross-validation technique was employed. The entire dataset was divided into 2 parts using cross-validation. The first one (training set) was to train the classifier. The second one (validation set) was used to find the hyperparameters of a classifier. The last one (testing set) was to evaluate the performance of the trained classifier. All the preprocessing steps were conducted in the training set. This included applying PCA to the linguistic features and eliminating redundant information among them [31]. An orthogonalization procedure was applied to the acoustic features to decorrelate them from the linguistic features. Both linguistic and acoustic features were then combined as an input for the machine learning classifier. The hyperparameters of the classifier are determined using the validation set. The trained classifier, together with the selected hyperparameters, was evaluated using the testing set. The detailed procedure is shown in Table S5 in Multimedia Appendix 1.

### Model Performance

All machine learning classifier models successfully distinguished stress levels based on the combination of linguistic and acoustic features (Table 1). Among them, the SVM-linear, SVM-sigmoid, SVM-RBF, and SVM-poly models outperformed the others, achieving ROC-AUC values of 78.28%, 78.77%, 76.65%, and 75.43%, respectively. The best-performing model, SVM-linear, not only achieved a high ROC-AUC value but also recorded the best *F*_1_-score and accuracy score, which were 75.27% and 73%, respectively.

**Table 1.** Performance of classifiers under combined linguistic and acoustic features.

Classifier	*F*_1_-score (SD), %	Accuracy (SD), %	ROC-AUC^a^ (SD), %
SVM-sigmoid^b^	71.67 (4.08)	56 (4.9)	78.77 (14.32)
SVM-linear^c^	75.27 (11.53)	73 (13.45)	78.28 (13.67)
SVM-RBF^d^	71.67 (4.08)	56 (4.9)	76.65 (14.16)
SVM-poly^e^	71.67 (4.08)	56 (4.9)	75.43 (20.85)
KNN^f^	70.83 (4.17)	57 (6.4)	63.34 (13.17)
Random forest	71.31 (3.93)	56 (4.9)	61.17 (18.28)
AdaBoost	71.67 (4.08)	56 (4.9)	59.66 (13.98)
ExtraTrees	71.31 (3.93)	56 (4.9)	52.47 (22.87)

a ROC-AUC: receiver operating characteristic-area under the curve.

b SVM-sigmoid: sigmoid kernel support vector machine.

c SVM-linear: linear support vector machine.

d SVM-RBF: radial basis function support vector machine.

e SVM-poly: polynomial support vector machine.

f KNN: k-nearest neighbor.

### Predictive Power of Feature Settings

In addition to the combined features, 2 additional feature settings, such as linguistic features only and acoustic features only, were applied to the machine learning training and testing. The ROC-AUC, *F*_1_-score, and accuracy values for the 4 best-performing classifier models across different feature settings are presented in Table 2. For the best-performing SVM-linear model, using linguistic features alone and acoustic features alone achieved ROC-AUC values of 76.68% and 60.92%, respectively. In contrast, the combination of linguistic and acoustic features achieved the highest ROC-AUC of 78.28%, demonstrating that the fusion analysis provides a clear predictive advantage. This pattern was consistent across other top SVM models (SVM-sigmoid, SVM-RBF, and SVM-poly). The results presented in Table 2 also reveal a performance imbalance in most models, where high *F*_1_-scores are not matched by similarly high accuracy. While this phenomenon appears in most models, it is not observed in the top-performing SVM-linear model. This discrepancy arises because most classifiers performed better for the high-stress status and were biased toward the TPR. In contrast, the SVM-linear model demonstrated a balanced performance across both high-stress and low-stress statuses, achieving strong results for both the TPR and the true negative rate.

**Table 2.** Performance of classifiers under different feature settings.

Classifier and feature setting	*F*_1_-score (SD), (%)	Accuracy (SD), (%)	ROC-AUC^a^ (SD), (%)
SVM-sigmoid^b^			
Combined (linguistic + acoustic)	71.67 (4.08)	56 (4.9)	78.77 (14.32)
Linguistic only	70.32 (11.35)	62 (13.27)	78.7 (13.02)
Acoustic only	70.01 (13.88)	60 (16.73)	57.58 (26.18)
SVM-linear^c^			
Combined (linguistic + acoustic)	75.27 (11.53)	73 (13.45)	78.28 (13.67)
Linguistic only	71.69 (16.28)	70 (16.12)	76.68 (17.19)
Acoustic only	56.95 (25.17)	54 (22)	60.92 (26.56)
SVM-RBF^d^			
Combined (linguistic + acoustic)	71.67 (4.08)	56 (4.9)	76.65 (14.16)
Linguistic only	71.69 (16.28)	70 (16.12)	76.68 (17.19)
Acoustic only	71.67 (4.08)	56 (4.9)	62.17 (21.8)
SVM-poly^e^			
Combined (linguistic + acoustic)	71.67 (4.08)	56 (4.9)	75.43 (20.85)
Linguistic only	72.82 (5.82)	61 (7)	75.15 (19.81)
Acoustic only	71.67 (4.08)	56 (4.9)	51.83 (26.45)

a ROC-AUC: receiver operating characteristic-area under the curve.

b SVM-sigmoid: sigmoid kernel support vector machine.

c SVM-linear: linear support vector machine.

d SVM-RBF: radial basis function support vector machine.

e SVM-poly: polynomial support vector machine.

### Importance of Acoustic Feature Processing

The acoustic features—used alongside linguistic features to achieve optimal predictive power—underwent an orthogonalization procedure to decorrelate them from the linguistic features. Given the natural correlation between these feature types in speech, this decorrelation process is essential to enhancing predictive performance. Such correlations can result in overlapping information when extracting features from the same audio recordings, which may lead to an overemphasis on the emotional state indicated by both feature types. To address this, we employed a novel orthogonalization procedure to ensure that the acoustic features were decorrelated from the linguistic features. Following this procedure, the acoustic features became orthogonal to the linguistic features, confirming successful decorrelation.

When the SVM-linear model was applied to the combined features without orthogonalization, it produced an ROC-AUC value of 57.22%—significantly lower than the optimal ROC-AUC value of 78.28% (Table 3). To verify the statistical significance, we compared the performance with and without orthogonalization using bootstrapping. We applied double cross-validation 100 times with different random seeds. We then computed the lower and upper CIs using the following formula.

**Table 3.** Performance of classifiers under combined features with or without processing the acoustic features through orthogonalization.

Classifier and orthogonalization	*F*_1_-score (SD), %	Accuracy (SD), %	ROC-AUC^a^ (SD), %
SVM-sigmoid^b^			
Yes	71.67 (4.08)	56 (4.9)	78.77 (14.32)
No	70.9 (14.39)	61 (17)	62.93 (23.43)
SVM-linear^c^			
Yes	75.27 (11.53)	73 (13.45)	78.28 (13.67)
No	60.95 (24.75)	60 (21.91)	57.22 (22.96)
SVM-RBF^d^			
Yes	71.67 (4.08)	56 (4.9)	76.65 (14.16)
No	69.26 (9.29)	60 (10.95)	60.9 (25.68)
SVM-poly^e^			
Yes	71.67 (4.08)	56 (4.9)	75.43 (20.85)
No	50.13 (21.19)	50 (17.32)	58.35 (27.53)

a ROC-AUC: receiver operating characteristic-area under the curve.

b SVM-sigmoid: sigmoid kernel support vector machine.

c SVM-linear: linear support vector machine.

d SVM-RBF: radial basis function support vector machine.

e SVM-poly: polynomial support vector machine.


(3) $$\begin{array}{r} \text{Evaluation metric with orthogonalization}\\ -\text{Metric evaluation without orthogonalization} \end{array}$$

The CIs for the 3 evaluation metrics are given in Table 4. The 2.5th percentile values for all 3 metrics are larger than 0, and thus, the performance with orthogonalization is better than without.

**Table 4.** CIs of the linear support vector machine (SVM-linear) model with and without processing the acoustic features through orthogonalization.

CIs	*F*_1_-score (%)	Accuracy (%)	ROC-AUC^a^ (%)
Lower confidence (2.5%)	9.7	7.22	5.57
Upper confidence (97.5%)	27.73	23.54	20.65

a ROC-AUC: receiver operating characteristic-area under the curve.

Similar results were observed with the other classifier models (SVM-sigmoid, SVM-RBF, and SVM-poly), underscoring the importance of processing acoustic features through orthogonalization in order to enhance classifier performance in predicting stress status.

### Stress Status Classification Performance

**Figure 4.**
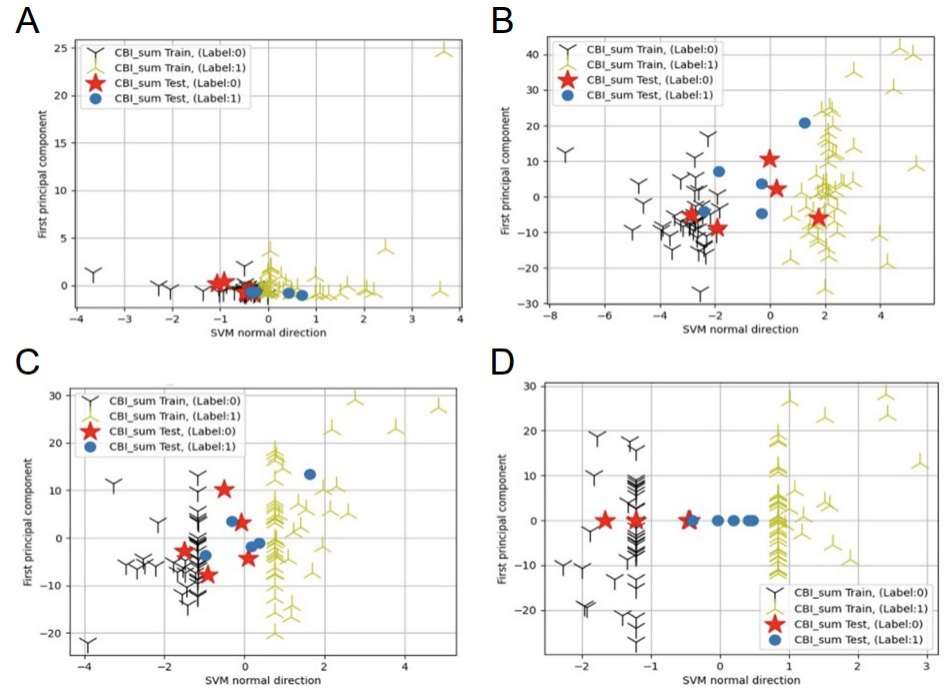
Performance with regard to classifying the stress status of testing samples using the linear support vector machine (SVM-linear) model under different feature settings. (A) Linguistic features only, (B) acoustic features only, (C) combined features without orthogonalization, and (D) combined features with orthogonalization. “Label: 0” represents the low-stress group and “Label: 1” represents the high-stress group. SVM: support vector machine.

The effectiveness of the best-performing SVM-linear model in classifying stress status in one of the testing sets of the 10-fold cross-validation is depicted in Figure 4. The SVM-linear model demonstrated only fair performance with regard to linguistic features alone (Figure 4A) or acoustic features alone (Figure 4B). Performance remained moderate even when combining features without processing the acoustic features through orthogonalization (Figure 4C). This indicates that proper feature processing, along with the fusion of linguistic and acoustic features in the SVM-linear model, provides strong predictive power for identifying individuals’ stress status based on their speech.

### Effects of Small Datasets

As our dataset is small, the proposed operations (using PCA and orthogonalization procedure) may affect the model’s performance. To verify this, we applied the SVM-linear model with double cross-validation 100 times with 100 different random seeds [39]. For each fold, we used the 3 evaluation measures: *F*_1_-score, accuracy, and ROC-AUC. The means and SDs of these 100 evaluation measures were reported in Table 5. It can be observed that the performances with the orthogonalization process are still above 70% for the 3 metrics. It is much better than the one without orthogonalization. Table 6 reports TPRt (also known as sensitivity), false positive rate, false negative rate, and true negative rate (also known as specificity), further justifying the reliability of the SVM-linear model.

**Table 5.** Performance of the linear support vector machine (SVM-linear) model under different feature settings and processing with double cross-validation 100 times with 100 different random seeds.

Feature setting	*F*_1_-score (SD), %	Accuracy (SD), %	ROC-AUC^a^ (SD), %
Combined (linguistic+acoustic) with orthogonalization	74.89 (1.83)	71.90 (1.83)	76.83 (3.65)
Combined (linguistic+acoustic) without orthogonalization	59 (4.17)	57.98 (3.69)	61.51 (3.92)
Linguistic only	71.84 (3.1)	69.5 (3.24)	75.29 (3.68)
Acoustic only	57.67 (2)	55 (1)	60.01 (2)

a ROC-AUC: receiver operating characteristic-area under the curve.

**Table 6.** Performance of the linear support vector machine (SVM-linear) model under different feature settings and processing with double cross-validation 100 times with 100 different random seeds.

Feature setting	TPR^a^ (SD), %	FPR^b^ (SD), %	FNR^c^ (SD), %	TNR^d^ (SD), %
Combined (linguistic + acoustic) with orthogonalization	76.48 (2.52)	33.52 (3.72)	23.52 (2.52)	66.48 (3.72)
Combined (linguistic + acoustic) without orthogonalization	57.17 (4.69)	40.36 (4.83)	42.83 (4.69)	59.64 (4.83)
Linguistic only	71.83 (3.84)	33.21 (5.2)	28.17 (3.84)	66.79 (5.2)
Acoustic only	59.33 (1)	50 (2)	40.67 (2)	50 (1)

a TPR: true positive rate.

b FPR: false positive rate.

c FNR: false negative rate.

d TNR: true negative rate.

## Discussion

This study introduced LASAP, a novel speech analytics machine learning program that, to our knowledge, is the first to successfully utilize a fusion analysis of linguistic and acoustic speech features to identify psychosocial stress among family caregivers in the Chinese community. Our results demonstrate that LASAP can effectively distinguish between individuals with high and low stress levels, particularly when using the SVM-linear classifier model, underscoring the significant potential of advanced speech analytics as an objective and scalable tool for psychosocial health assessment.

The robustness of the LASAP approach was evident, with 4 classifier models (SVM-linear, SVM-sigmoid, SVM-RBF, and SVM-poly) achieving ROC-AUC values greater than 75%. The SVM-sigmoid and SVM-linear models were the top 2 performers, with ROC-AUC values of 78.77% and 78.28%, respectively. While the SVM-sigmoid model yielded a slightly higher ROC-AUC value than that of the SVM-linear model, a deeper examination of other performance metrics reveals a more nuanced picture. The strong performance on additional metrics, particularly high *F*_1_-score and accuracy, is crucial, as these indicate the model’s precision and recall in identifying individuals under stress. The SVM-sigmoid model’s lower *F*_1_-score (71.67%) and accuracy (56%) suggest that it was biased toward correctly identifying the high-stress state, potentially at the expense of misclassifying low-stress individuals. In contrast, the SVM-linear model demonstrated a more balanced performance, with a superior *F*_1_-score (75.27%) and accuracy (73%). In psychosocial health-screening applications, where the goal is to accurately identify those who need support and those who do not, this balanced performance is critical. Therefore, the SVM-linear model’s consistent superiority across all 3 metrics establishes it as the most robust and reliable classifier for practical applications.

The strong performance of the LASAP with the SVM-linear model has significant implications for addressing the well-documented challenges in psychosocial condition management. With 75% of individuals in need not receiving adequate intervention [3] and the WHO reporting that only 25% of the member states have integrated mental health services into primary health care [40], the need for scalable solutions is urgent. However, traditional psychosocial health assessments are the major bottleneck as they are time-consuming, require specialist administrators, can be subjective, and are vulnerable to clients’ self-report bias [8,9]. By providing an objective, automated, and effective alternative, LASAP directly targets these limitations. Its ability to analyze speech from a brief, nonsensitive interview could make it a practical tool for widespread screening, helping to identify at-risk individuals who can then be referred for further support.

The efficiency gains offered by LASAP are substantial. Our data collection based on 30-minute interviews using 12 general questions (Table S1 in Multimedia Appendix 1) is considerably shorter than the 1 to 2 hours often required for conventional assessments utilizing multiple instruments. This efficiency, coupled with its automated and objective nature, positions LASAP as a tool that could significantly enhance the accessibility of psychosocial screening. Its use of nonsensitive questions is a key strength as it mitigates the response bias, which is a common limitation of traditional self-reports, by allowing participants to express themselves naturally [8]. For a vulnerable population, such as family caregivers, this provides a less intrusive and more accurate assessment of their psychosocial well-being.

Another key contribution of this study is the comparative analysis using linguistic features, acoustic features, and their combination in the assessment of stress. Previous research has often focused on either linguistic or acoustic features in isolation. In our study, the combination of these features yielded significantly improved results, with the SVM-linear model achieving an ROC-AUC value of 78.28% when both types of features were analyzed together. In contrast, the use of linguistic features alone resulted in an ROC-AUC value of 76.68%, while acoustic features alone achieved an ROC-AUC value of only 60.92%. Similar improvements were observed in the high-performing models, including SVM-sigmoid, SVM-RBF, and SVM-poly. These findings clearly demonstrate that the fusion of linguistic and acoustic features enhances the model’s predictive power, supporting the hypothesis that a multifaceted approach to speech analytics can lead to more accurate assessments of psychosocial stress. This not only confirms the value of feature fusion but also suggests that future research should explore combined feature sets more extensively, which may advance model robustness and enhance applications in real-world scenarios.

Furthermore, our study indicated that the proper processing of acoustic features is pivotal to the effectiveness of the LASAP. Our study implemented an orthogonalization procedure to decorrelate acoustic features from linguistic features before combining them for analysis. This step is critical in reducing the risk of overfitting, which can occur when redundant information is present in the data. Emotional intensity conveyed by both linguistic and acoustic features can create a misleadingly strong signal if not managed properly. The results indicate the necessity of this orthogonalization process for decorrelation. When the SVM-linear model was applied to combined features without decorrelation, the ROC-AUC value dropped dramatically to 57.22%, a stark contrast to the optimal ROC-AUC value of 78.28% achieved with orthogonalized features. Similar results were observed in the SVM-sigmoid, SVM-RBF, and SVM-poly models. This indicates that the proper processing of acoustic features can greatly reduce overfitting and improve the accuracy and reliability of stress status classification. These findings point to a broader significance in the application of machine learning techniques in our field, suggesting that enhanced feature processing could be vital for other models and datasets to bolster model performance.

The implications of this study extend beyond the immediate findings. The successful application of the LASAP in identifying stress among family caregivers opens avenues for broader applications in various populations experiencing psychosocial stressors. Future research should explore the applicability of this model in diverse settings, including psychosocial health screenings in workplaces, educational institutions, and community health initiatives. Moreover, the insights gained from this study can inform the development of more sophisticated automated systems that integrate multiple features and ensure proper processing. As psychosocial conditions continue to rise globally, the demand for accessible, efficient, and accurate assessment tools becomes increasingly urgent. The LASAP exemplifies how the integration and proper processing of features can provide accurate assessments, facilitating timely interventions and support for individuals in need.

While the results are promising, this study has limitations. The sample size of 100 family caregivers, though adequate for preliminary findings, may not fully capture the diversity of experiences among different caregiver populations. Future studies should aim for larger, more diverse samples to validate the findings and enhance generalizability. Additionally, exploring the influence of demographic factors, such as age, gender, and cultural background, on speech characteristics could provide deeper insights into the psychosocial stress assessment [41]. Moreover, while our focus was on stress identification, future research could also investigate the potential of the LASAP in assessing other psychosocial conditions, such as anxiety and depression, through similar fusion analyses. The model’s adaptability could pave the way for a comprehensive suite of tools addressing various aspects of psychosocial health.

This study highlights the significant potential of integrating linguistic and acoustic features for the automated assessment of psychosocial stress. The excellent performance of the SVM-linear model reinforces the importance of using combined features and proper processing techniques, such as orthogonalization, to enhance predictive accuracy. As we move toward an era of digital health solutions, the findings from the LASAP can serve as a foundation for developing innovative tools that improve mental health assessments and interventions, ultimately contributing to better psychosocial outcomes for individuals across various contexts.

## Supplementary material

10.2196/91138 Multimedia Appendix 1 Illustration of the orthogonalization procedure, interview questions, caregiver stress-indicating words and topics related to Walsh’s family resilience theory, description of acoustic features, hyperparameters of different machine learning classifiers, and implementation details of the proposed algorithm.

## Abbreviations

ASAP: Automatic Speech Analytic Program
CBI: Caregiver Burden Inventory
COMPARE: Computational Paralinguistics Challenge
LASAP: Linguistic and Acoustic Speech Analytics Program
LLD: low-level descriptor
PCA: principal component analysis
ROC-AUC: receiver operating characteristic-area under the curve
SVM: support vector machine
SVM-linear: linear support vector machine
SVM-poly: polynomial support vector machine
SVM-RBF: radial basis function support vector machine
SVM-sigmoid: sigmoid kernel support vector machine
TPR: true positive rate
WHO: World Health Organization
