# Supplementary material for: Psychosocial Stress in the Chinese Community: Speech Analytics Through Linguistic and Acoustic Fusion Using Machine Learning
Source: JMIR Biomed Eng. 2026 May 29;11:e91138. doi: 10.2196/91138 (PMC13221159; doi:10.2196/91138)
Supplement: Multimedia Appendix 1 [file biomedeng-v11-e91138-s001.docx]

**Identifying Psychosocial Stress in the Chinese Community: The Power of Linguistic and Acoustic Fusion in Speech Analytics through Machine Learning**

| **Supplementary Figure 1. Illustration of the orthogonalization procedure.**   \| 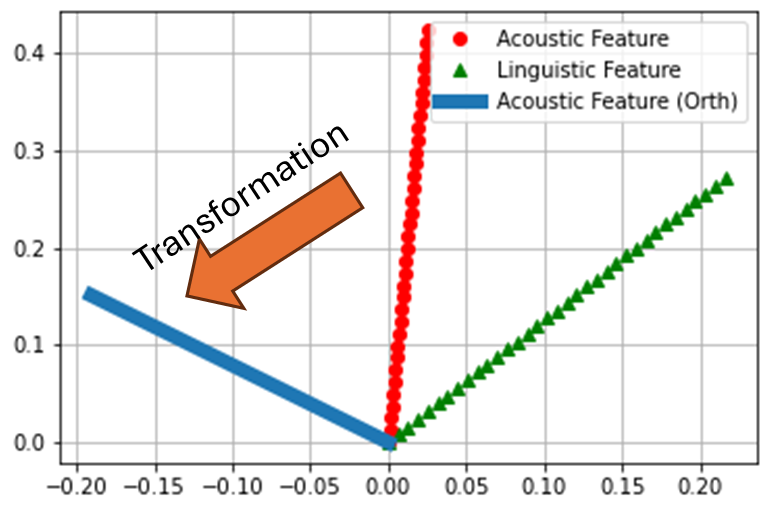 \| \| --- \| \|  \|   **Supplementary Table 1**  Interview Questions   \| Family Belief System  What make you stressful?  When you feel bad, what you will do to make yourself feel better?  What does it mean by adversity according to your experience?  What keeps you persevering in the face of adversity? \| \| --- \| \| Organizational Pattern  Who is on your mind when you feel weak and why?  How many new friends did you make at the Centre*#*?  Do you think making new friends are helpful to you?  Is there anything make you feel deeply at the Centre? \| \| Communicational Pattern  What troubles you the most when taking care of your family members?  Who do you talk to when you are unhappy and why?  Who in your family motivates you the most and why?  What gives you the most satisfaction in caring for your family members? \|   *# the non-profit organization participated in this study*  **Supplementary Table 2. Caregiver Stress-Indicating Words and Topics Related to Walsh’s Family Resilience Theory.** | | | | | |
| --- | --- | --- | --- | --- | --- | --- | --- | --- | --- | --- |
|  | **Topic** | **Word** | **Total Word Count** | **Low-Stress** | **High-Stress** |
| **Family Belief System** | 健康與醫療  (Health and Medical Care) | 睇醫生 (See a Doctor) | 3 | 0 | 3 |
|  |  | 有病 (Sick) | 5 | 4 | 1 |
|  |  | 照顧 (Care) | 249 | 150 | 99 |
|  | 住房與生活環境 (Housing and Living Environment) | 劏房 (Subdivided Flat) | 2 | 2 | 0 |
|  |  | 困住 (Trapped) | 4 | 3 | 1 |
|  | 人際關係 (Interpersonal Relationships) | 離婚 (Divorce) | 3 | 3 | 0 |
|  |  | 分居 (Separation) | 3 | 3 | 0 |
|  | 方法與嘗試  (Methods and Attempts) | 方法 (Method) | 42 | 33 | 9 |
|  |  | 嘗試 (Attempt) | 5 | 4 | 1 |
| **Organizational pattern** | 教育與學習  (Education and Learning) | 成績 (Grades) | 2 | 2 | 0 |
|  |  | 補習社 (Tutoring Center) | 2 | 2 | 0 |
|  |  | 老師 (Teacher) | 5 | 4 | 1 |
|  |  | 功課 (Homework) | 3 | 3 | 0 |
|  |  | 讀書 (Studying) | 11 | 8 | 3 |
|  |  | 考試 (Exams) | 9 | 9 | 0 |
|  | 個人特質  (Personal Traits) | 堅持 (Persistence) | 101 | 64 | 37 |
|  |  | 固執 (Stubbornness) | 2 | 0 | 2 |
|  | 動力與價值觀  (Motivation and Values) | 值得 (Worthwhile) | 5 | 2 | 3 |
|  |  | 原動力 (Motivation) | 64 | 31 | 33 |
|  | 宗教與信仰  (Religion and Belief) | 宗教 (Religion) | 3 | 0 | 3 |
|  |  | 信仰 (Faith) | 2 | 0 | 2 |
|  |  | 祈禱 (Prayer) | 3 | 3 | 0 |
|  |  | 教會 (Church) | 3 | 2 | 1 |
|  | 娛樂與休閒  (Entertainment and Leisure) | 聽歌 (Listen to Music) | 4 | 1 | 3 |
|  |  | 玩下 (Play a bit) | 10 | 3 | 7 |
|  |  | 行街 (Shopping) | 8 | 3 | 5 |
|  |  | 買嘢 (Buy things) | 5 | 3 | 2 |
|  |  | 打機 (Play Games) | 3 | 3 | 0 |
| **Communication Pattern** | 語言與文化 (Language and Culture) | 國語 (Mandarin) | 3 | 0 | 3 |
|  |  | 身份證 (Identity Card) | 8 | 7 | 1 |
|  |  | 廣東話 (Cantonese) | 2 | 0 | 2 |
|  |  | 新移民 (New Immigrants) | 4 | 4 | 0 |
|  |  | 香港 (Hong Kong) | 22 | 18 | 4 |
|  |  | 大陸 (Mainland China) | 10 | 6 | 4 |
|  | 兒童與家庭 (Children and Family) | 小朋友 (Children) | 186 | 103 | 83 |
|  |  | 仔仔 (Boy) | 4 | 1 | 3 |
|  |  | 小孩 (Kid) | 2 | 2 | 0 |
|  |  | 阿仔 (Son) | 27 | 8 | 19 |
|  | 溝通與交流 (Communication and Interaction) | 傾傾 (Chat) | 3 | 3 | 0 |
|  |  | 傾偈 (Talk) | 3 | 3 | 0 |
|  |  | 傾計 (Converse) | 66 | 35 | 31 |
|  |  | 打畀 (Call) | 4 | 3 | 1 |
|  |  | 傾下 (Talk a bit) | 36 | 22 | 14 |
|  |  | 搵邊個 (Find someone) | 5 | 1 | 4 |
|  |  | 傾訴 (Confide) | 12 | 9 | 3 |
|  | 人際關係與合作 (Interpersonal Relationships and Cooperation) | 融洽 (Harmony) | 2 | 1 | 1 |
|  |  | 溝通 (Communication) | 19 | 13 | 6 |
|  | 心理與情感 (Psychology and Emotions) | 信心 (Confidence) | 3 | 3 | 0 |
|  |  | 希望 (Hope) | 29 | 17 | 12 |
|  |  | 緊張 (Nervousness) | 11 | 6 | 5 |
|  |  | 脆弱 (Vulnerability) | 64 | 44 | 20 |
|  |  | 脾氣 (Temper) | 12 | 5 | 7 |
|  |  | 壓力 (Stress) | 109 | 51 | 58 |

**Supplementary Table 3. Description of acoustic features.**

| Category | Subset | Number of subsets | Description |
| --- | --- | --- | --- |
| Pitch and voicing | F0 | 1 | It measures the pitch of the voice. |
|  | VoicingFinalUnclipped | 1 | This is a confidence score (ranging from 0 to 1) for “is this sound voiced?”. |
| Voice quality | jitterLocal | 1 | It measures how much your voice pitch moves from one sound wave to the next. For example, voice shakiness. |
|  | jitterDDP | 1 | It measures how quickly the voice pitch changes. For example, they track if your voice shakiness is getting faster or slower. |
|  | shimmerLocal | 1 | It measures how much your voice loudness changes between sound waves. For example, sudden volume jumps. |
|  | logHNR | 1 | It measures voice clarity versus breathiness. High HNR indicates clear voice (for example, an opera singer), while low HNR indicates breathy/hoarse voice (for example, a whisper). |
| Spectral features | pcm_fftMag | 15 | It shows which pitches (frequencies) are present in your voice. It can be considered as the “fingerprint” of your voice. |
|  | MFCC | 14 | This is a compact “voice ID code” used for recognizing speakers or words. It can be considered as the “passwords” of your voice. |
|  | audspec | 26 | It measures how your voice sounds to human ears. |
|  | audspecRasta | 1 | A feature created by cleaning up “audspec” with background noises ignored. |
|  | sum_audspec | 1 | It measures the total power of your voice over time. |
| Energy | pcm_RMSenergy | 1 | It measures the average loudness of your voice. |
|  | pcm_zcr | 1 | It detects noisy versus tonal sounds. A high pcm_zcr value indicates whispers or consonants (for example, “s”), while a low pcm_zcr value indicates vowels (for example, “a”). |
| Subtotal | | 65 |  |

| **Supplementary Table 4. Hyperparameters of different machine learning classifiers.** | |
| --- | --- |
| **Classifier Name** | **Parameters** |
| Ada-Boost | 'n_estimators':[10,100,500,1000],'learning_rate':[0.001,0.01,0.1,1] |
| ExtraTrees | 'n_estimators':[10,100,500,1000],'criterion':['gini','entropy’],  'min_samples_split':[2,4,6,8] |
| KNN | n_neighbors = 1,3,5,7,9 |
| Random Forest | 'n_estimators':[10,100,500,1000],'criterion':['gini','entropy’],  'min_samples_split':[2,4,6,8] |
| SVM-poly | 'gamma':['scale','auto'],'C':[1e-2,1e-1,1,3,5,7,10,20,50] |
| SVM-rbf | 'gamma':['scale','auto'],'C':[1e-2,1e-1,1,3,5,7,10,20,50] |
| SVM-sigmoid | 'gamma':['scale','auto'],'C':[1e-2,1e-1,1,3,5,7,10,20,50] |
| SVM-linear | ‘penalty’: ['l1','l2’], ,'C':[1e-2,1e-1,1,3,5,7,10,20,50] |

**Supplementary Table 5. Implementation details of the proposed algorithm.**

| For each training set: |
| --- |
| Linguistic Features  Step 1.1: Count the occurrence of the selected 53 words.  Step 1.2: Apply PCA to the word counts and obtain the salient linguistic features (i.e. the counts) by retaining 99% of the total variance.  Step 1.3: Apply QR factorization to the salient linguistic features and obtain the orthogonal matrix. The orthogonal matrix will be used to perform orthogonalization.  Acoustic Features  Step 2.1: Extract all the acoustic features.  Step 2.2: Apply the orthogonal matrix to the acoustic features and obtain enhanced acoustic features. The orthogonalization procedure can remove acoustic information that are similar to the linguistic features.  Step 2.3: Find the means and standard deviations of the enhanced acoustic features for low-stress and high-stress classes. Apply unpaired t-test formula to each enhanced acoustic features and output the statistics. Enhanced acoustic features are selected as the salient acoustic features if the statistic is larger than 1.645 (i.e. 95% confidence interval).  Combining the features  Step 3.1 Combine salient linguistic features and salient acoustic features.  Step 3.2 Feed the combined features to the classifier. |

| For each testing set: |
| --- |
| Linguistic Features  Step 1.1: Count the occurrence of the selected 53 words.  Step 1.2: Apply the learnt PCA from the training set to the word counts and obtain the salient linguistic features (i.e. the counts).  Acoustic Features  Step 2.1: Extract all the salient acoustic features (features selected in Step 2.3, the training phase).  Step 2.2: Apply the learnt orthogonal matrix to the salient acoustic features and obtain uncorrelated acoustic features.  Combining the features  Step 3.1 Combine salient linguistic features and uncorrelated acoustic features.  Step 3.2 Feed the combined features to the classifier. |
